# Precision fluid therapy in sepsis: fluid responsiveness vs. fluid tolerance

**DOI:** 10.3389/fmed.2026.1980813

**Published:** 2026-10-05

**Authors:** Ali Araghi

**Affiliations:** Georgia College of Medicine, Augusta University, Augusta, GA, United States

**Keywords:** critical care ultrasound, fluid responsiveness, fluid tolerance, passive leg raise, pulse pressure variation, septic shock, stroke volume, venous congestion

## Abstract

Intravenous fluid is the most frequently administered drug in septic shock and one of the least precisely dosed. Two decades of trials have progressively dismantled the assumption that more fluid is better: protocolized early goal-directed therapy failed to improve on contemporary usual care, high-volume bolus protocols caused frank harm where organ support was limited, and restrictive strategies applied after an initial resuscitation have proven neutral rather than superior. What has replaced a universal volume prescription is a pair of questions that must be asked separately for each patient and repeated over time: will this patient's cardiac output actually rise with more fluid (fluid responsiveness), and can the lungs, kidneys, and venous system absorb that volume without harm (fluid tolerance)? This narrative review examines how to answer both questions in the modern intensive care unit. We ground the discussion in Guytonian venous return physiology and in the revised Starling model, in which sepsis-induced glycocalyx degradation makes infused crystalloid a short-acting drug with a cumulative side-effect profile. Using an index case of a mechanically ventilated patient with pneumococcal septic shock, atrial fibrillation, lung-protective tidal volumes, obesity, and acute kidney injury, we show why the two most widely used bedside indices, respiratory variation in inferior vena cava diameter and pulse pressure variation, are physiologically invalid in precisely the patients in whom the fluid decision is hardest, and why maintenance infusions and fluid creep deserve the same scrutiny as boluses. We make the case for direct stroke volume measurement paired with a reversible dynamic challenge, most commonly a passive leg raise, as the one approach that remains interpretable across arrhythmia, low tidal volume, spontaneous respiratory effort, and elevated intra-abdominal pressure, and we discuss dynamic arterial elastance as a complementary tool for predicting whether arterial pressure will follow flow. We review the outcome evidence, including the FRESH trial and supporting meta-analyses, the emerging literature on sepsis endotypes and subphenotypes, and the 2026 Surviving Sepsis Campaign recommendations. Finally, we address fluid tolerance and de-resuscitation, where venous congestion assessment and lung ultrasound are increasingly used, and outline where the evidence remains thin.

## 1 Introduction

Few interventions in critical care are as reflexive as the fluid bolus, and few are as poorly individualized. A liter of crystalloid is ordered with less deliberation than a dose of vancomycin, despite comparable potential for organ injury. The 2026 Surviving Sepsis Campaign (SSC) guidelines continue to suggest at least 30 mL/kg of intravenous crystalloid within the first three hours for sepsis-induced hypoperfusion, but they do so as a conditional recommendation supported by low-certainty evidence, and they explicitly instruct clinicians to reassess frequently, to individualize the volume to the patient in front of them, and to use adjusted or ideal body weight when body mass index exceeds 30 kg/m^2^ (1). That last remark alone reframes the bundle from a fixed prescription into a starting estimate.

Beyond that initial estimate, the guidelines offer no volume target. For patients who remain hypoperfused after 30 mL/kg, the SSC panel could recommend neither a liberal nor a restrictive strategy, citing wide protocol heterogeneity across trials (1). The panel did recommend a change in method rather than dose: for adults with sepsis or septic shock, dynamic measures should guide fluid resuscitation rather than physical examination or static measures alone (1).

In comparison, the European Society of Intensive Care Medicine (ESICM) addresses early volume management in its 2025 Clinical Practice Guideline on Fluid Therapy in Adult Critically Ill Patients (Part 2: Volume of Resuscitation Fluids). The ESICM suggests administering up to 30 mL/kg of intravenous crystalloids during the initial phase for adult patients with sepsis or septic shock, rather than mandating a minimum amount; this conditional recommendation is supported by very low-certainty evidence. Unlike the Surviving Sepsis Campaign, which historically emphasized a minimum of at least 30 mL/kg, the ESICM frames this volume as an upper reference cap, allowing clinicians to tailor the volume downward as needed (2).

The clinical question has therefore shifted from “how much fluid?” to “how do I know whether this particular bolus will help or harm this particular patient right now?”

To address this question, this narrative review is organized around a single case that captures most of the physiological traps encountered in real intensive care practice, and it argues that the answer requires two independent assessments: responsiveness and tolerance, measured by tools that remain valid under the conditions in which critically ill patients are actually ventilated and monitored.

## 2 The index case

A 55-year-old man is admitted to the medical ICU with severe pneumonia and septic shock. He was intubated in the emergency department and has received 30 mL/kg of lactated Ringer's solution per ideal body weight (using the Devine formula). He is on norepinephrine at 0.35 µg/kg/min. Heart rate is 110 in atrial fibrillation, blood pressure is 95/60 mmHg (mean arterial pressure 72 mmHg), temperature is 37.5 °C, and oxygen saturation is 88%. He is ventilated in assist-control mode with a set rate of 20 and a total rate of 29, tidal volume 450 mL (6.35 mL/kg ideal body weight), PEEP 12 cmH_2_O, and FiO_2_ 1.0. Body mass index is 37 kg/m^2^. Creatinine is 4.25 mg/dL, blood urea nitrogen 38 mg/dL, and white cell count 18,300/µL. The chest radiograph shows bilateral airspace opacities. He remains hypotensive on vasopressor support with rising creatinine and worsening oxygenation. (Figure 1) The bedside team must decide how to guide any further intravenous fluid. Four strategies are commonly proposed: titrating to changes in inferior vena cava (IVC) diameter, titrating to changes in stroke volume, titrating to pulse pressure variation (PPV), or simply running maintenance lactated Ringer's at 100 mL/h. Each carries a different physiological assumption, and in this patient only one survives scrutiny.

**Figure 1:**
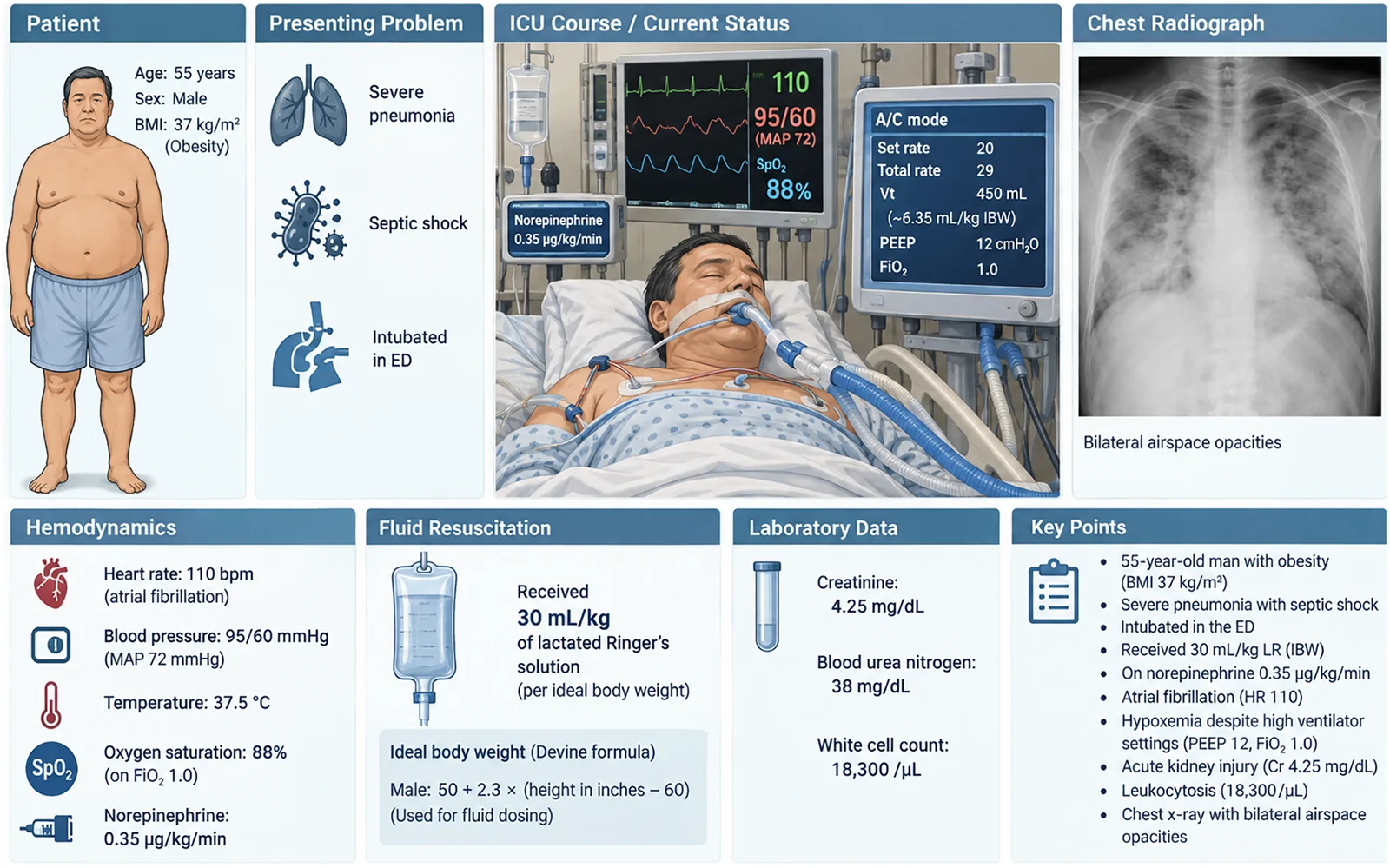
Clinical snapshot of the index case. The combination of atrial fibrillation, lung-protective tidal volume, spontaneous respiratory effort above the set rate, obesity, and acute kidney injury is common in septic shock and, as discussed below, invalidates most conventional indices of fluid responsiveness.

### 2.1 Basic physiology and hemodynamic concepts

To manage fluid administration, it is essential to understand the difference between blood pressure and blood flow. The goal of fluid therapy is to optimize tissue perfusion, not blood pressure readings. Blood pressure is the product of cardiac output and arterial vascular resistance, while blood flow in circulation depends on the pressure gradient between upstream mean systemic filling pressure in veins (mSPF) and upstream right atrial pressure (RAP).

#### 2.1.1 Guytonian framework: venous return and cardiac output

Arthur Guyton's model describes the circulation as a closed-loop system in which cardiac output and venous return must be equal at steady state. The relationship between the venous return curve and the ventricular Starling curve helps explain how changes in vascular filling, right atrial pressure, and ventricular contractility determine the circulation's operating point (3).

##### 2.1.1.1 Venous return curve

Figure 2 plots venous return against right atrial pressure (RAP). As RAP falls, the pressure gradient between mean systemic filling pressure (mSFP) and the right atrium increases, promoting greater venous return and, consequently, higher cardiac output. Conversely, a higher RAP increases downstream resistance to venous return and reduces the pressure gradient driving blood back to the heart.

**Figure 2:**
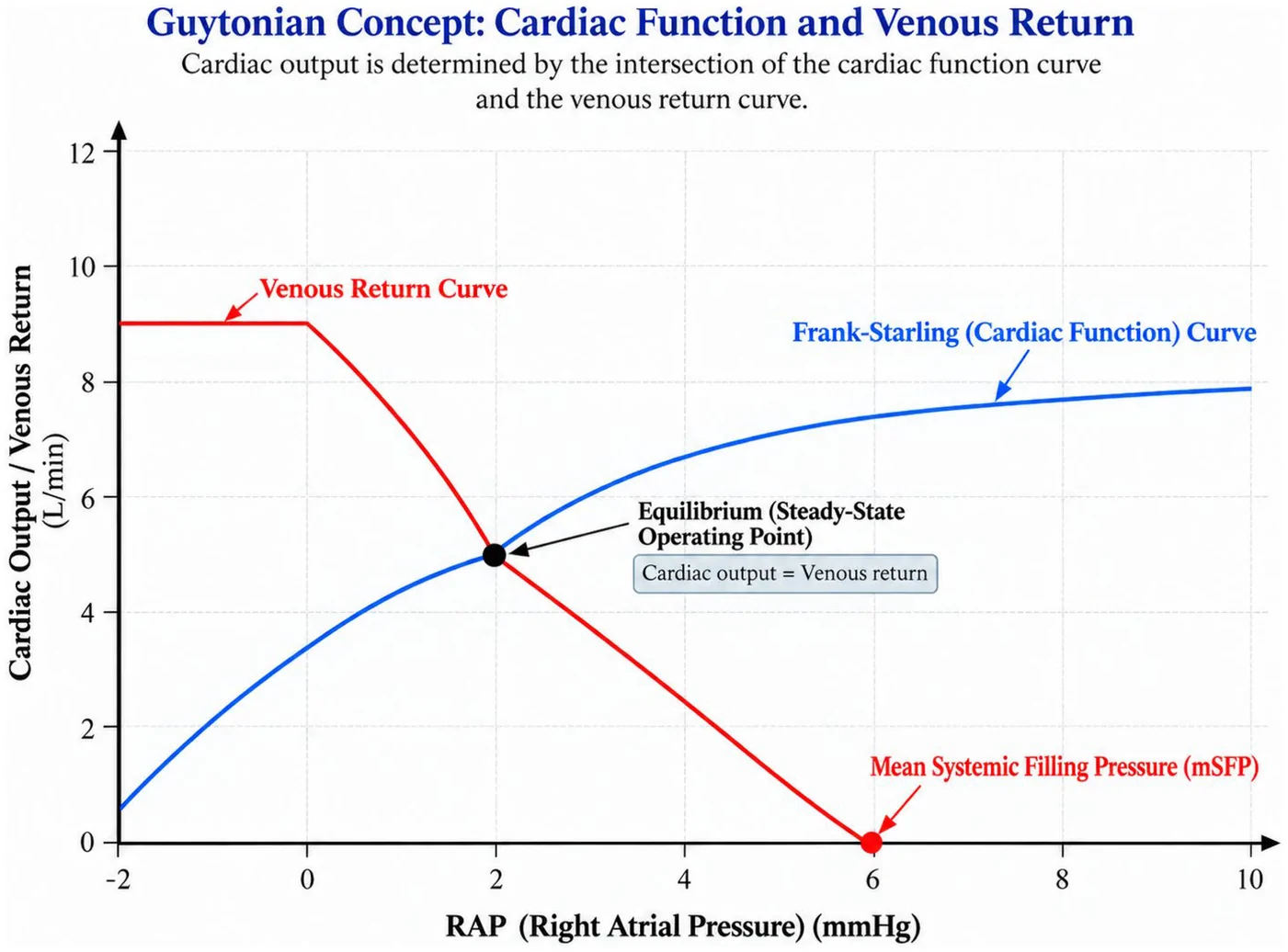
Superimposition of venous flow/cardiac output and right atrial pressure.

#### 2.1.2 Equilibrium point

The intersection of the venous return curve and the ventricular Starling curve represents the circulatory equilibrium point, where venous return equals cardiac output. This point shifts when preload, vascular tone, blood volume, intrathoracic pressure, or ventricular contractility changes.

Role of Contractility and Respiratory Pressure

- Greater right or left ventricular contractility lowers RAP and increases venous return.
- Reduced contractility raises RAP and limits venous return.
- Respiratory intrapleural pressure swings also influence RAP and therefore affect venous return.

Clinically, this framework is useful for fluid management because it emphasizes that fluid administration increases cardiac output only when it meaningfully raises stressed volume and venous return without excessively increasing filling pressures. This concept also explains why passive leg raising (PLR) is a diagnostic maneuver to determine whether the patient is fluid responsive. PLR redirects the patient's venous reserve toward the right atrium and can increase stroke volume only if the patient's cardiac performance is on the steep part of the Frank-Starling curve. Likewise, vasopressors like norepinephrine improve stroke volume not only by increasing cardiac contractility but also by improving venous return through alpha-1 receptor agonism.

## 3 Why fluid is not a benign intervention

### 3.1 The degraded glycocalyx and the revised starling principle

The classical Starling model, in which capillary filtration is opposed by an interstitial oncotic pressure and fluid is reabsorbed at the venular end, has been superseded. In the revised model, the endothelial glycocalyx, a gel-like layer of proteoglycans and glycosaminoglycans lining the luminal endothelial surface, creates a subglycocalyx space whose oncotic pressure, not that of the interstitium, governs transcapillary flux (4). Under physiological conditions, venular reabsorption is minimal or absent; filtered fluid returns via lymphatics.

Sepsis degrades this layer. Inflammatory mediators, matrix metalloproteinases, heparanase, and tumor necrosis factor shed glycocalyx constituents into the circulation, and fragments such as syndecan-1 correlate with severity and mortality (5). This markedly increases effective vascular permeability, so infused crystalloid distributes preferentially into the interstitium rather than expanding the intravascular compartment (Figure 3). Rapid, high-volume infusion compounds the problem: acute hypervolemia releases atrial natriuretic peptide, which itself sheds the glycocalyx (6). Fluid loading in sepsis therefore partly destroys the barrier that would have retained it.

**Figure 3:**
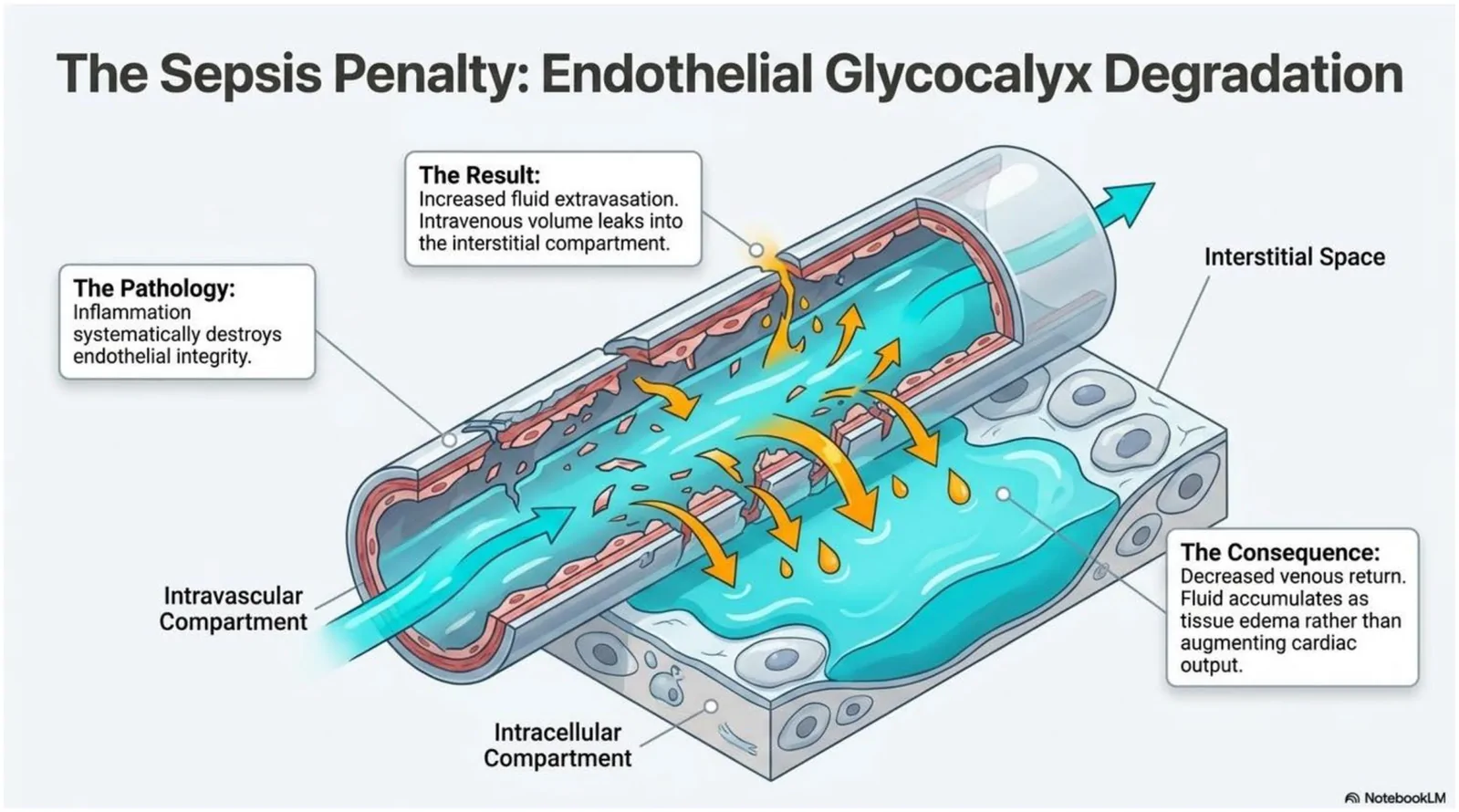
Endothelial glycocalyx degradation in sepsis. Loss of endothelial barrier integrity increases extravasation of infused volume into the interstitium, so that a given crystalloid dose produces less intravascular expansion and more tissue edema. Adapted from the concepts described by Woodcock and Woodcock (4) and Uchimido et al. (5).

The clinical corollary has been demonstrated directly. In patients with circulatory shock who responded to a fluid challenge, the hemodynamic benefit had largely dissipated within 60 min (7, 8). In an ovine endotoxemia model, fluid resuscitation transiently raised cardiac output but worsened glycocalyx shedding and did not improve the underlying shock state (9). Fluid in sepsis is a short-acting drug with a cumulative, long-lasting side-effect profile.

### 3.2 Cumulative balance and organ injury

Observational data across cohorts consistently link positive cumulative fluid balance to mortality in sepsis, independent of illness severity (10, 11). Mechanistically, this is unsurprising. Interstitial edema in the lung worsens gas exchange and prolongs mechanical ventilation, as the FACTT trial established in acute lung injury, where a conservative strategy increased ventilator-free day (12). In the kidney, venous congestion raises renal interstitial and subcapsular pressure, reducing the transrenal perfusion gradient, which explains why volume expansion in an already congested patient can worsen rather than improve oliguria.

This has given rise to the four-phase model of fluid therapy, rescue, optimization, stabilization, and evacuation (the ROSE concept), in which the goals and acceptable fluid balance differ fundamentally by phase (13, 14). The bolus that is appropriate in the first hour of rescue may be actively harmful in the same patient forty-eight hours later.

### 3.3 Two questions, not one

A fluid administration decision requires two separate determinations (Figure 4). Fluid responsiveness asks a purely cardiac question: is the ventricle operating on the steep portion of the Frank–Starling curve, such that an increase in preload will translate into a meaningful increase in stroke volume? Fluid tolerance asks a downstream question: even if cardiac output rises, can the lungs, kidneys, and venous circulation accommodate the added volume without causing pulmonary edema, venous congestion, or worsening acute kidney injury?

**Figure 4:**
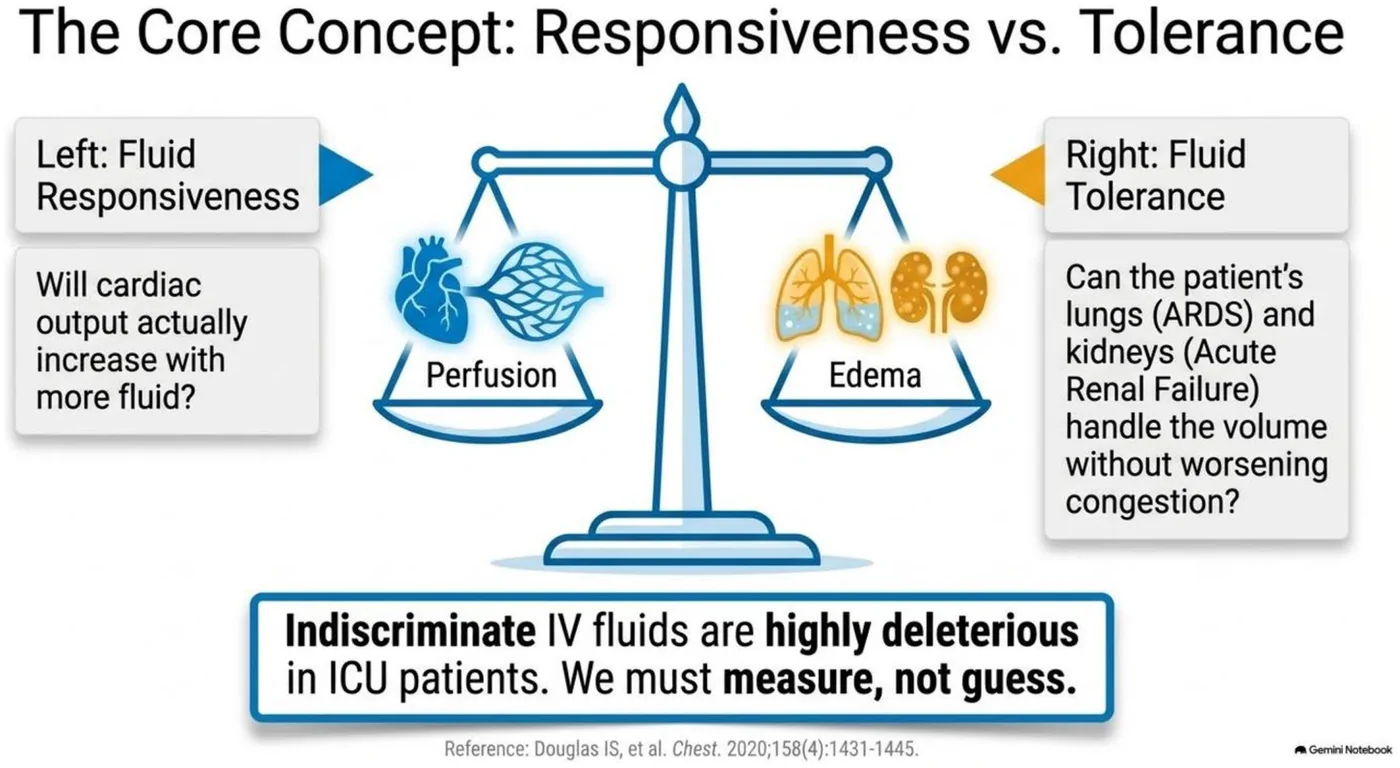
Fluid responsiveness and fluid tolerance are independent axes. A patient may be responsive but intolerant; in that case, the appropriate action is vasopressor escalation rather than volume. Only patients who are both responsive and tolerant should receive further fluid.

These axes are independent, and the combinations matter. A responsive but intolerant patient—arguably the description of our index case, with bilateral infiltrates, PEEP 12, FiO_2_ 1.0, and a creatinine of 4.25 mg/dL—is one in whom further volume may raise cardiac output at an unacceptable cost to gas exchange and renal venous pressure. In that setting, escalating vasopressor support and accepting a somewhat lower cardiac output may be the better trade. A non-responsive patient should not receive fluid at all, regardless of tolerance. Roughly half of hemodynamically unstable ICU patients are non-responders at any given moment, which means that, on average, a policy of empirical bolusing is a coin flip.

## 4 What the landmark trials actually established

Any argument for individualized fluid therapy has to be reconciled with a large and largely neutral trial literature. The tables below summarize the four questions those trials addressed: resuscitation strategy, volume, fluid type, and perfusion target (See Tables 1–4).

**Table 1:** Resuscitation strategy and early goal-directed therapy.

Trial	Population	Intervention vs control	Key outcome
Rivers (2001) (15)	Severe sepsis/septic shock, single-centre ED	6 h EGDT (CVP, MAP, ScvO_2_ targets) vs standard care	Marked reduction in in-hospital mortality; basis for early SSC bundles
ProCESS (2014) (16)	1,341 septic shock, 31 US sites	EGDT vs protocol-based standard vs usual care	60-day mortality 21.8% vs 18.2% vs 18.9% (*P* = 0.83); no benefit of EGDT
ARISE (2014) (17)	1,600 septic shock, Australia/NZ	EGDT vs usual care	90-day mortality 18.6% vs 18.8% (*P* = 0.90); no benefit
ProMISe (2015) (18)	1,260 septic shock, 56 UK sites	EGDT vs usual care	90-day mortality 29.5% vs 29.2% (*P* = 0.90); no benefit

**Table 2:** Volume: restrictive versus liberal strategies.

Trial	Population	Intervention vs control	Key outcome
FEAST (2011) (28)	3,141 children, febrile illness, Africa	Saline or albumin bolus vs no bolus	Increased 48 h mortality with any bolus (RR: 1.45; 95% CI: 1.13–1.86)
SSSP-2/Andrews (2017) (29)	209 sepsis with hypotension, Zambia	Aggressive fluid bundle (∼3.5 L/6 h) vs usual care	Increased in-hospital mortality 48.1% vs 33.0% (*P* = 0.03)
CLASSIC (2022) (30)	1,554 septic shock, Europe	Restrictive vs standard IV fluid after initial resuscitation	90-day mortality 42.3% vs 42.1% (*P* = 0.46); neutral
CLOVERS (2023) (31)	1,563 sepsis-induced hypotension, US	Restrictive (vasopressor-first) vs liberal fluids	90-day mortality 14.0% vs 14.9% (*P* = 0.61); neutral, halted for futility
ARISE FLUIDS (2026) (32)	1,000 ED septic shock, Australia/NZ	Restricted fluid with early vasopressors vs greater fluid, later vasopressors	No difference in days alive and out of hospital at day 90; ∼1.1 L less fluid in the vasopressor arm

**Table 3:** Fluid type: crystalloid composition and colloid.

Trial	Population	Intervention vs control	Key outcome
SMART (2018) (33)	15,802 ICU (1,641 sepsis), US	Balanced crystalloids vs 0.9% saline	Reduced MAKE30 overall (14.3% vs 15.4%, *P* = 0.04); sepsis 30-day mortality 26.3% vs 31.2% (*P* = 0.01)
BaSICS (2021) (34)	10,520 ICU (1,987 sepsis), Brazil	Balanced vs saline	90-day mortality 26.4% vs 27.2% (*P* = 0.47); higher mortality in traumatic brain injury subgroup
PLUS (2022) (35)	5,037 ICU, Australia/NZ	Balanced vs saline	90-day mortality 21.8% vs 22.0%; no difference
ALBIOS (2014) (36)	1,818 severe sepsis/septic shock	Albumin plus crystalloid vs crystalloid	No 28- or 90-day mortality benefit
SAFE (2004) (37)	6,997 heterogeneous ICU (sepsis subgroup)	4% albumin vs saline	No overall mortality difference

**Table 4:** Perfusion targets and personalized, dynamic resuscitation.

Trial	Population	Intervention vs control	Key outcome
ANDROMEDA-SHOCK (2019) (38)	424 early septic shock, Latin America	Capillary refill time- vs lactate-guided resuscitation	28-day mortality 34.9% vs 43.4% (*P* = 0.06); less fluid given with CRT guidance
FRESH (2020) (39)	124 septic shock, 13 US/UK sites	PLR/stroke-volume-guided vs usual care	Lower net fluid balance at 72 h; less renal replacement therapy (5.1% vs 17.5%) and mechanical ventilation (17.7% vs 34.1%), both *P* = 0.04

### 4.1 Resuscitation strategy and early goal-directed therapy

The lesson of ProCESS, ARISE, and ProMISe is not that hemodynamic monitoring is useless. It is that a rigid protocol anchored to static targets—central venous pressure in particular—adds nothing to attentive contemporary care. Meta-analyses show central venous pressure has essentially no ability to discriminate fluid responders from non-responders, with an area under the curve close to 0.56 (19). A protocol built on an uninformative variable cannot be expected to outperform clinical judgment.

### 4.2 Volume: restrictive vs. liberal strategies

Advancements in molecular biology and data analysis have provided new pathophysiological frameworks for assessing fluid volume strategies, informed by sepsis endotypes and subphenotypes.

Sepsis endotypes represent biologically distinct, molecularly defined subgroups, primarily derived from whole-blood transcriptomics, distinguishing them from clinical subphenotypes that rely on downstream bedside variables and standard biomarkers (20). The primary molecular framework organizes patients along an axis of hyperinflammation vs. immunosuppression and adaptive immune competence. Major independent transcriptomic classifications include the SRS scheme (SRS1 denoting immunosuppression and higher mortality; SRS2 denoting immunocompetence), the Mars groups (Mars1 through Mars4, with Mars1 featuring severe innate/adaptive suppression and the worst prognosis), and the Stanford system (inflammopathic, adaptive, and coagulopathic clusters) (21, 22). These approaches have combined into unified models, such as the three Consensus Transcriptomic Subtypes (CTS1–3) and the 4-cluster Hi-DEF framework. These models group patient profiles into categories of myeloid and lymphoid dysregulation vs. protective immune responses (23, 24).

At the bedside, simple immunological tests help classify patients into actionable groups. For example, some patients show macrophage activation-like syndrome (MALS), which can be treated with IL-1 blockers. Others display immunoparalysis, characterized by reduced monocyte HLA-DR expression or low lymphocyte counts (21).

Clinical systems like the *α*–*β*–*γ*–*δ* phenotypes can predict outcomes using routine data (25). Additionally, subtypes based on endothelial dysfunction markers, such as syndecan-1 and angiopoietin-2, are especially good at predicting which patients will respond differently to treatment, particularly in determining who is harmed by excessive fluids vs. who can tolerate volume better (26, 27).

However, applying these findings in practice is still difficult. Challenges include inconsistent results across molecular data types, slow test turnaround times, and changes in patient biology during ICU stays (20).

Based on the trials above, two conclusions follow, and they point in different directions. First, protocolized high-volume resuscitation causes harm when pushed well beyond 50–70 mL/kg, particularly when mechanical ventilation and vasopressor support are not readily available (28, 29). Second, once an initial one to three liters have been given, further restriction confers no mortality advantage (30–32). Critically, in CLASSIC, CLOVERS, and ARISE FLUIDS, almost every patient received substantial fluid before randomization, so the safety of a genuinely low-volume strategy from the outset remains untested. These trials also compared two fixed strategies; none tested whether titrating volume to the individual patient's physiology outperforms either.

### 4.3 Fluid type: crystalloid composition and colloid

The 2026 SSC guidelines recommend crystalloids as first-line fluid (strong recommendation, moderate certainty), suggest balanced crystalloids over 0.9% saline (conditional, moderate certainty) with an explicit exception for traumatic brain injury, suggest crystalloid alone over crystalloid plus supplemental albumin (conditional, moderate certainty), and recommend against starches (strong, high certainty) (1). For most septic patients, the composition question is settled well enough; the dosing question is not.

### 4.4 Perfusion targets and personalized dynamic resuscitation

These two trials differ from the rest in kind, not degree. Neither prescribed a volume. Both prescribed a method of deciding, patient by patient and hour by hour, whether the next intervention should be fluid or something else. Both reduced fluid delivery, and both showed benefit (See Tables 5 and 6).

**Table 5:** Summary of the trials evidence.

Synthesis of the trial evidence
Protocolized EGDT anchored to static targets is not superior to modern usual care (ProCESS, ARISE, ProMISe).
High-volume protocols beyond 50–70 mL/kg cause harm, especially where organ support is limited (FEAST, SSSP-2).
After initial resuscitation, restrictive and liberal strategies yield similar mortality (CLASSIC, CLOVERS, ARISE FLUIDS); very-low-volume strategies remain untested.
Balanced crystalloids are favored over 0.9% saline, except in traumatic brain injury (SSC 2026, conditional recommendation).
Perfusion- and dynamically guided titration reduces fluid load and organ support and may reduce mortality (ANDROMEDA-SHOCK, FRESH).

**Table 6:** Clinical take-home points.

Clinical take-home points
Indiscriminate intravenous fluid actively harms patients with ARDS and acute kidney injury; maintenance infusions and fluid creep contribute at least as much cumulative volume as resuscitation boluses.
Atrial fibrillation, tidal volume below 8 mL/kg, elevated intra-abdominal pressure, and spontaneous respiratory effort invalidate PPV and IVC-based assessment. Only about 2% of ICU patients satisfy all PPV validity criteria at any given time.
Stroke volume, averaged over multiple cycles, remains reliable despite arrhythmia and does not depend on heart–lung interaction.
Stroke volume must be paired with a reversible dynamic challenge—preferably a passive leg raise performed according to the five rules—with a threshold of roughly 10%.
Responsiveness and tolerance are separate questions. A responsive but intolerant patient should receive vasopressors, not volume.
Dynamically guided resuscitation reduces net fluid balance, renal replacement therapy, and mechanical ventilation, and is a conditional recommendation in the 2026 Surviving Sepsis Campaign guidelines.

## 5 Why conventional bedside indices fail in real patients

Every dynamic index carries physiological prerequisites. The problem is not that these indices are invalid in principle; it is that their prerequisites are routinely violated in the modern ICU, most often in the patients whose fluid status is hardest to judge.

In addition, it is crucial to understand the relationship between improving flow and tissue perfusion vs. blood pressure (MAP). In patients with dominant vasoplegia, they may respond to a fluid challenge by increasing cardiac output without increasing MAP. Thus, trying to increase MAP by providing more volume or increasing vasopressor dosage may cause more harm than benefit.

To determine which group of hypotensive patients is expected to increase MAP after a fluid challenge, measurement of arterial dynamic elastance can be useful.

Dynamic arterial elastance (Eadynamic) is a functional, beat-to-beat index of ventriculo-arterial coupling defined as the ratio of pulse pressure variation to stroke volume variation (PPV/SVV) during controlled mechanical ventilation (40–43). Rather than representing static arterial elastance, it reflects dynamic heart-lung interactions and quantifies how efficiently a cyclic change in stroke volume is converted into pulse pressure across the arterial tree (40, 42, 44, 45). Clinically, its primary purpose is not to identify preload responsiveness, a role fulfilled by PPV or SVV alone, but to determine whether arterial blood pressure will increase after volume administration in a patient known to be preload-dependent (40, 42, 46). Measurement requires an arterial line and either pulse contour analysis, calibrated transpulmonary thermodilution, or transthoracic echocardiography (such as LVOT VTI or Delta Vpeak) to capture simultaneous variations (41–44).

In clinical practice, an Eadynamic > 0.73–0.9 in hypotensive, fluid-responsive patients reliably predicts an increase in mean arterial pressure (MAP) after volume expansion, whereas a low value indicates vasoplegia, in which added flow fails to generate perfusion pressure (40, 42, 46). Beyond fluid administration, an Eadynamic > 1.0 (or >0.8 by echocardiographic assessment) identifies vasopressor-dependent individuals who can tolerate norepinephrine weaning without rebound hypotension (40, 43). Despite its utility, the metric has important limitations: it is unvalidated in outcome-based interventional trials and shares the limitations of dynamic cardiopulmonary indices, making it inaccurate during spontaneous breathing, cardiac arrhythmias, low tidal volume ventilation (<8\mL/kg), or altered intra-abdominal and thoracic mechanics (40, 45, 47, 48).

### 5.1 Inferior vena cava diameter and its respiratory variation

Respiratory variation in IVC diameter is attractive because it is fast, non-invasive, and requires no arterial line. Its validity, however, rests on the assumption that changes in IVC caliber across the respiratory cycle reflect changes in right atrial pressure driven by a known swing in pleural pressure, in a vessel with normal compliance. Three conditions must be met: fully passive ventilation, a tidal volume large enough to generate an adequate pleural pressure swing (8 mL/PBW), and normal intra-abdominal pressure. Our index case violates all three (Figure 5).

**Figure 5:**
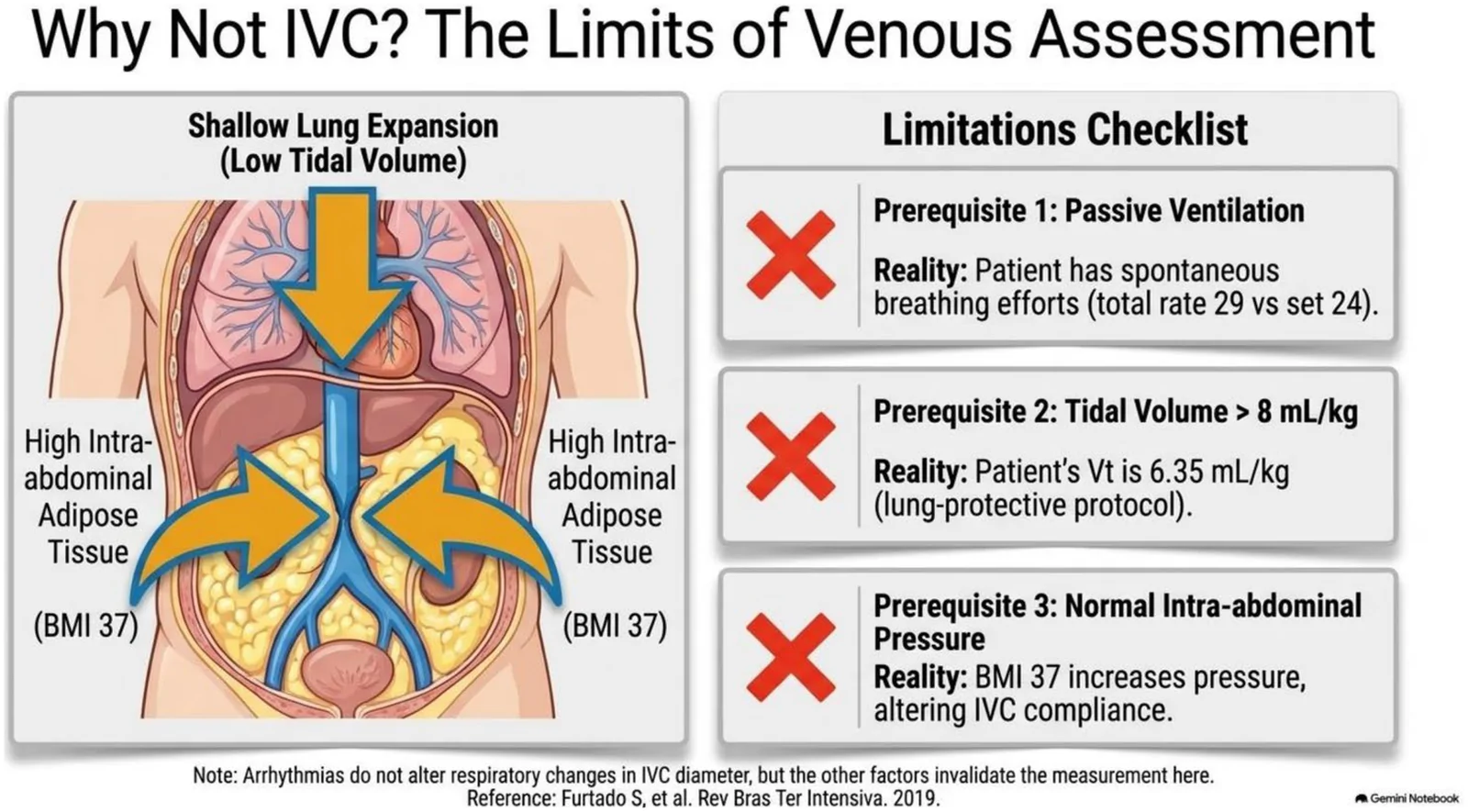
Why IVC diameter measurement is unreliable in the index case. Spontaneous respiratory effort (total rate 29 above a set rate of 24), lung-protective tidal volume (6.35 mL/kg), and obesity-related elevation in intra-abdominal pressure (BMI 37) each independently invalidate the measurement. Adapted from Furtado and Reis (49).

Spontaneous effort is the most immediate problem. Negative intrathoracic pressure from patient effort collapses the IVC regardless of volume status, producing spuriously high collapsibility. A tidal volume of 6.35 mL/kg, mandated by lung-protective ventilation, generates insufficient pleural pressure swing to distend the vessel measurably, producing false negatives. Obesity also raises intra-abdominal pressure, which alters transmural pressure across the intrahepatic and infrahepatic IVC and shifts its compliance curve, so a given diameter no longer maps predictably onto right atrial pressure (49, 50).

These are not theoretical concerns. In a prospective comparison of echocardiographic indices in ventilated patients, IVC-derived measures performed poorly for predicting fluid responsiveness, with substantially lower discrimination than left ventricular outflow tract velocity–time integral (LVOT VTI) variation during a preload challenge (51). Via and colleagues cataloged ten distinct situations in which IVC ultrasound fails, most of which are routine in a medical ICU (50). Of note, arrhythmia is not among them: atrial fibrillation does not by itself invalidate respiratory IVC variation. In this patient, the other three violations are sufficient.

### 5.2 Pulse pressure variation

PPV has a stronger physiological pedigree and better published performance than IVC indices, but its prerequisites are even more restrictive. Valid application requires controlled mechanical ventilation with no spontaneous effort, a regular cardiac rhythm, a tidal volume of at least 8 mL/kg ideal body weight, a heart rate to respiratory rate ratio above 3.6, respiratory system compliance above 30 mL/cmH_2_O, and no significant right ventricular dysfunction (52).

Atrial fibrillation is disqualifying in a way that deserves emphasis. PPV infers preload dependence from the cyclic modulation of stroke volume by the respiratory cycle. In atrial fibrillation, stroke volume varies substantially from beat to beat because of variable diastolic filling times, and this variation has nothing to do with heart–lung interaction. The measured PPV is real but uninterpretable; it is noise masquerading as signal (Figure 6).

**Figure 6:**
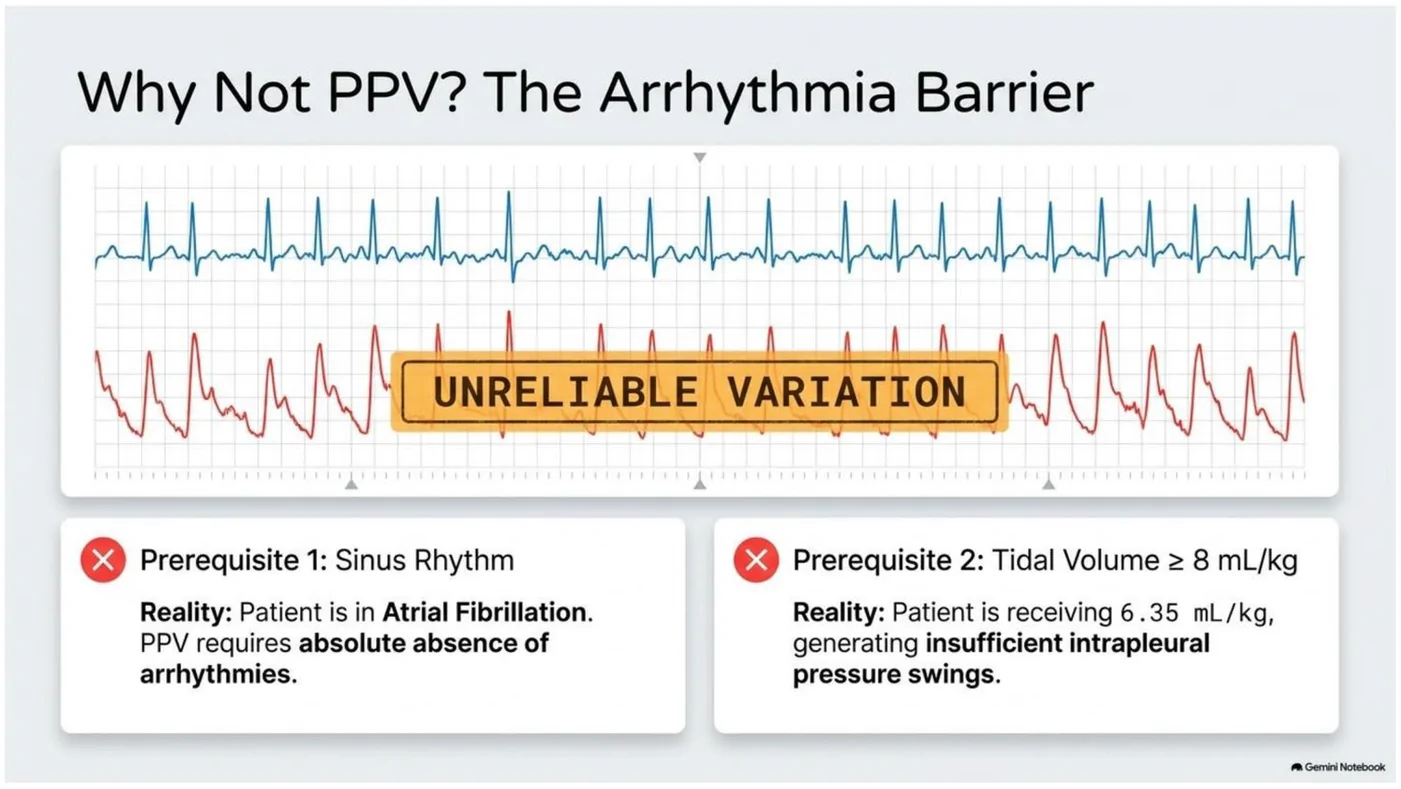
Why PPV fails in the index case. Atrial fibrillation produces beat-to-beat variation in stroke volume that is unrelated to heart–lung interaction, and a tidal volume of 6.35 mL/kg generates insufficient cyclic change in pleural pressure. Either violation alone is disqualifying.

Low tidal volume is the second violation. De Backer and colleagues showed that PPV loses predictive value below 8 mL/kg because the pleural pressure swing becomes too small to unmask preload dependence, generating false negatives in patients who would in fact respond (53, 54). Since lung-protective ventilation at 6 mL/kg is a strong SSC recommendation with high-certainty evidence for sepsis-associated ARDS (1), the conditions for valid PPV and the conditions for good ventilator management are in direct conflict.

Mahjoub and colleagues best capture the magnitude of this problem in a single-day point-prevalence study across 26 French ICUs. Of 311 patients, only six (2%) met all validity criteria for PPV. Even restricting the analysis to the 170 patients with an arterial line already in place, only five, or 3%, qualified (52). PPV is an excellent tool in the operating theatre, where patients are paralyzed, in sinus rhythm, and ventilated at conventional tidal volumes. It is applicable to a small minority of medical ICU patients at any given moment.

There are workarounds worth knowing. The tidal volume challenge, transiently increasing tidal volume from 6 to 8 mL/kg for one minute and measuring the change in PPV or stroke volume variation, restores predictive accuracy under lung-protective ventilation (55). It does not, however, rescue PPV in atrial fibrillation.

### 5.3 Maintenance fluid and the problem of fluid creep

The fourth option in our case—maintenance lactated Ringer's at 100 mL/h—deserves separate treatment because it is the option most often chosen by default and least often examined. Van Regenmortel and colleagues quantified the contribution of non-resuscitation fluid in a tertiary mixed ICU cohort and found that maintenance fluids and fluid creep, meaning the volume delivered as drug diluents, keep-vein-open infusions, and flushes, imposed a larger cumulative fluid, sodium, and chloride burden than resuscitation fluids themselves (56) (Figure 7).

**Figure 7:**
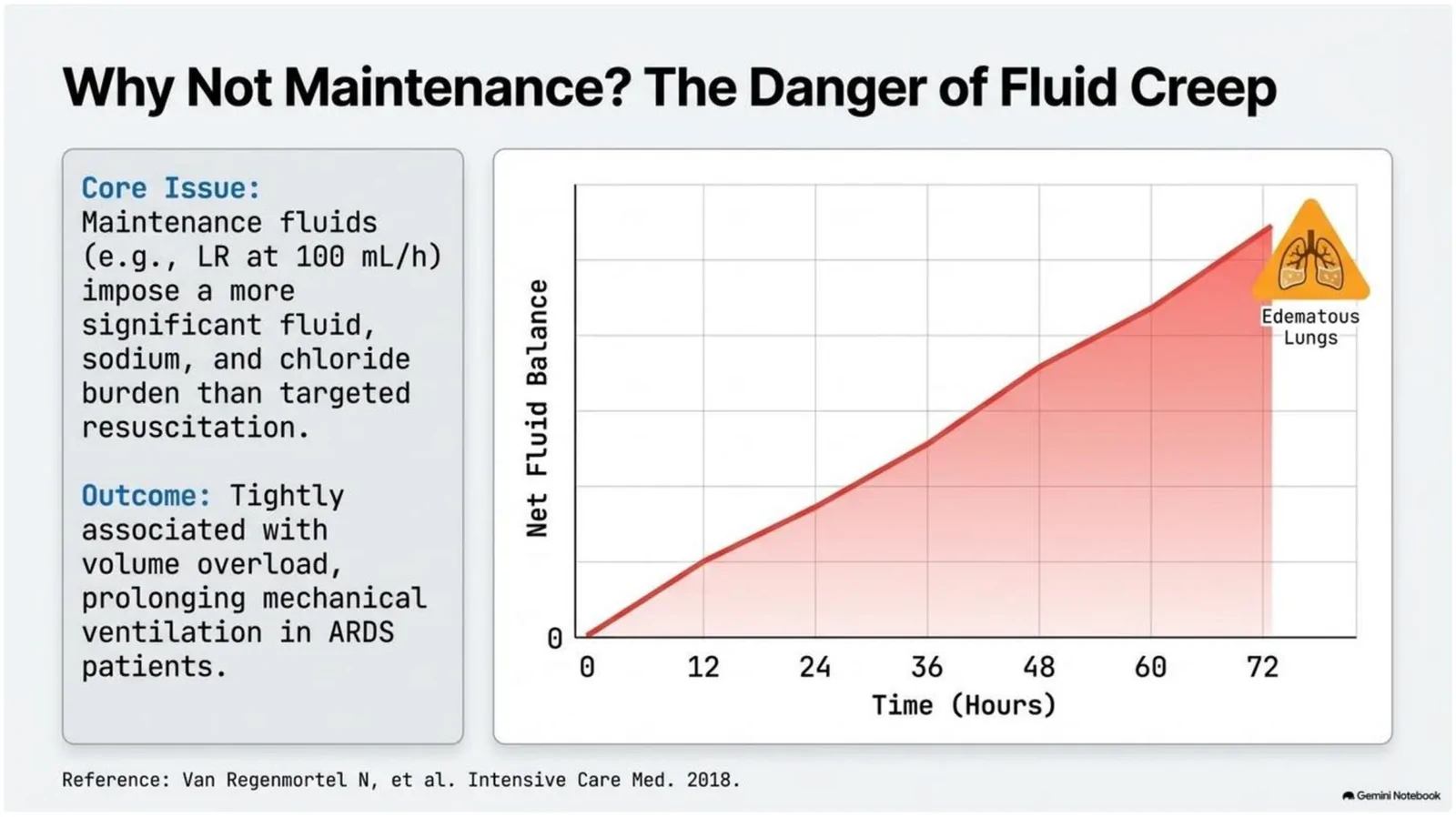
Fluid creep. A continuous maintenance infusion of 100 mL/h delivers 2.4 L per day and roughly 7.2 L over 72 h, independent of any resuscitation boluses. In a patient with ARDS and acute kidney injury, this cumulative burden is closely associated with prolonged mechanical ventilation. Adapted from Van Regenmortel et al. (56).

The arithmetic is simple and rarely performed at the bedside: 100 mL/h is 2.4 L per day and over 7 L across a 72 h ICU stay, delivered to a patient who is anuric, hypoxemic, and already 35 mL/kg positive. In a patient with ARDS and acute kidney injury, this is not a neutral background infusion. It is an unmonitored resuscitation delivered slowly.

### 5.4 Matching the tool to the physiology

Figure 8 summarizes the applicability of the three candidate monitoring approaches against the four physiological features of our index case. Stroke volume paired with a dynamic challenge is the only modality that remains valid across all four.

**Figure 8:**
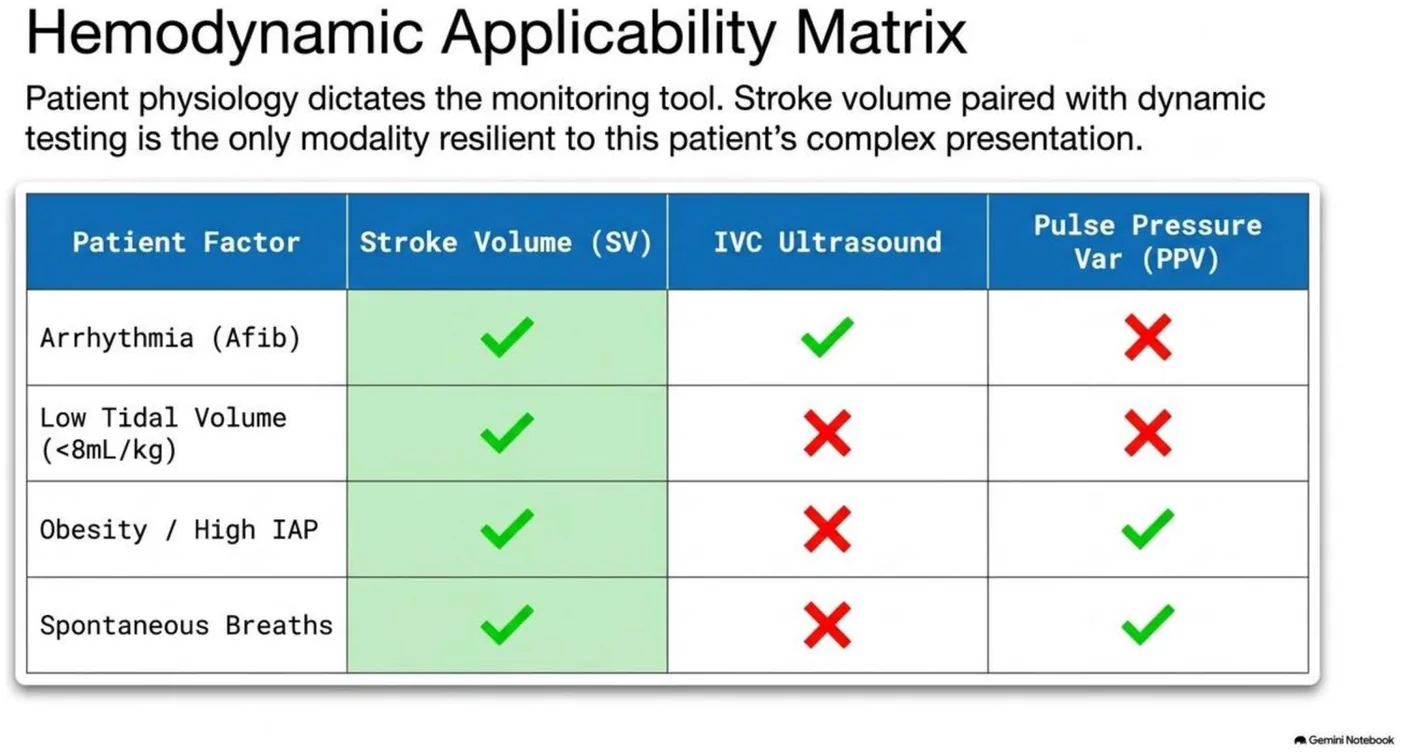
Applicability matrix. Patient physiology, rather than institutional habit or equipment availability, should determine the monitoring modality. Stroke volume combined with a reversible preload challenge is the only approach that survives arrhythmia, low tidal volume, obesity with raised intra-abdominal pressure, and spontaneous respiratory effort simultaneously.

## 6 Stroke volume: the resilient signal

### 6.1 Why stroke volume survives where the indices fail

The advantage of stroke volume is conceptual before it is technical. PPV and IVC variation are surrogates: they infer preload dependence from the way a respiratory perturbation modulates a downstream signal, and they therefore inherit every assumption embedded in that inference. Stroke volume is the output variable itself. When it is measured before and after a preload challenge, no assumption about heart–lung interaction is required.

This is why arrhythmia, which is fatal to PPV, is manageable for stroke volume. Individual beats vary in atrial fibrillation, but the average stroke volume over enough consecutive cycles is stable and reproducible. Convention is to average across at least five to ten beats, and more in the presence of marked irregularity (57). The beat-to-beat variability that destroys PPV is simply averaged away (Figure 9).

**Figure 9:**
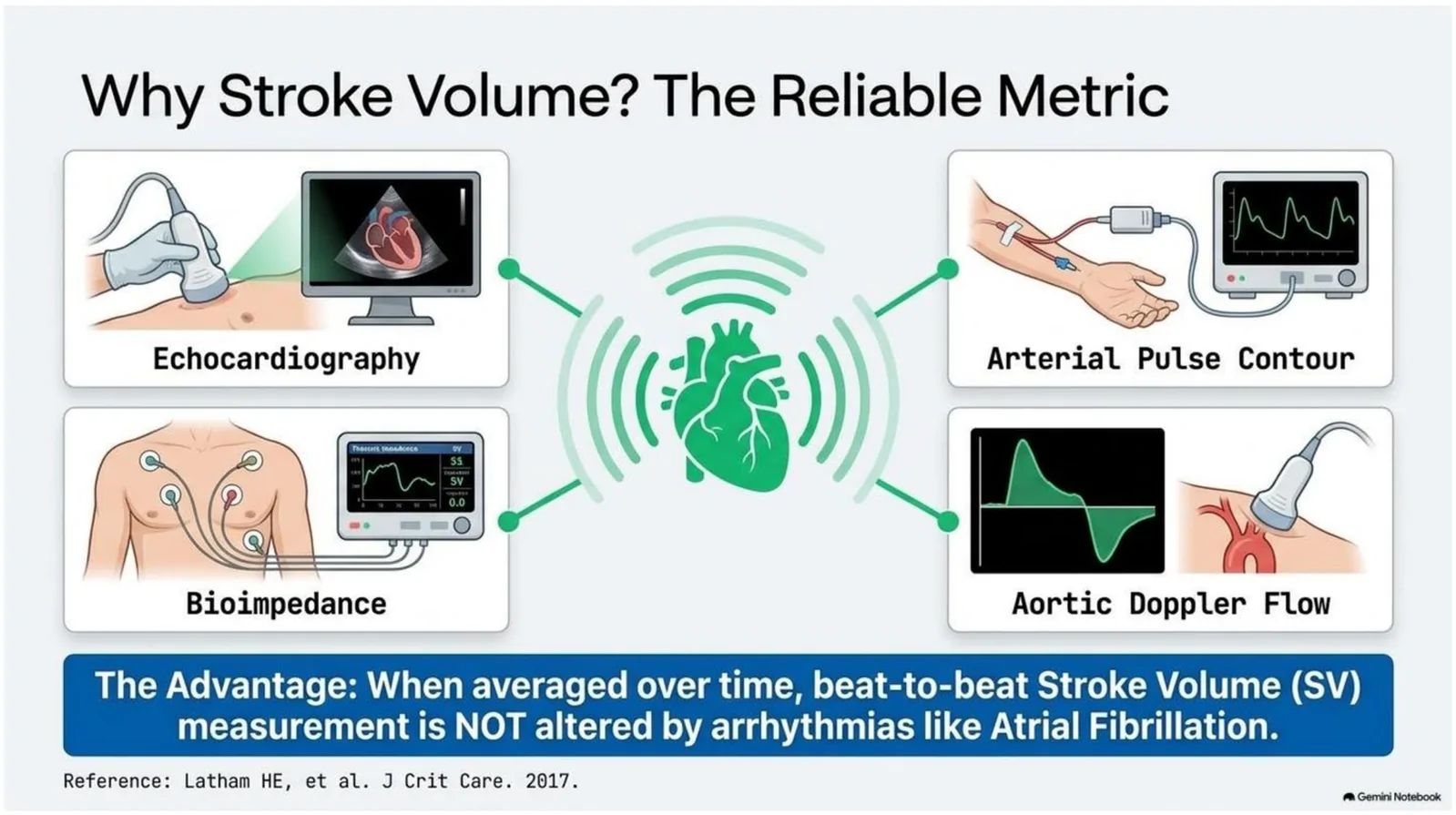
Modalities for measuring stroke volume at the bedside: transthoracic or transesophageal echocardiography, arterial pulse contour analysis, thoracic bioimpedance or bioreactance, and esophageal aortic Doppler. Each has a distinct profile of invasiveness, operator dependence, and validity under arrhythmia.

### 6.2 Measuring stroke volume by the LVOT VTI method

For most intensivists, echocardiographic LVOT VTI is the most accessible and most robust technique, requiring no additional catheter or proprietary monitor. Stroke volume is the product of the cross-sectional area of the left ventricular outflow tract and the velocity–time integral of blood traversing it (Figure 10).

**Figure 10:**
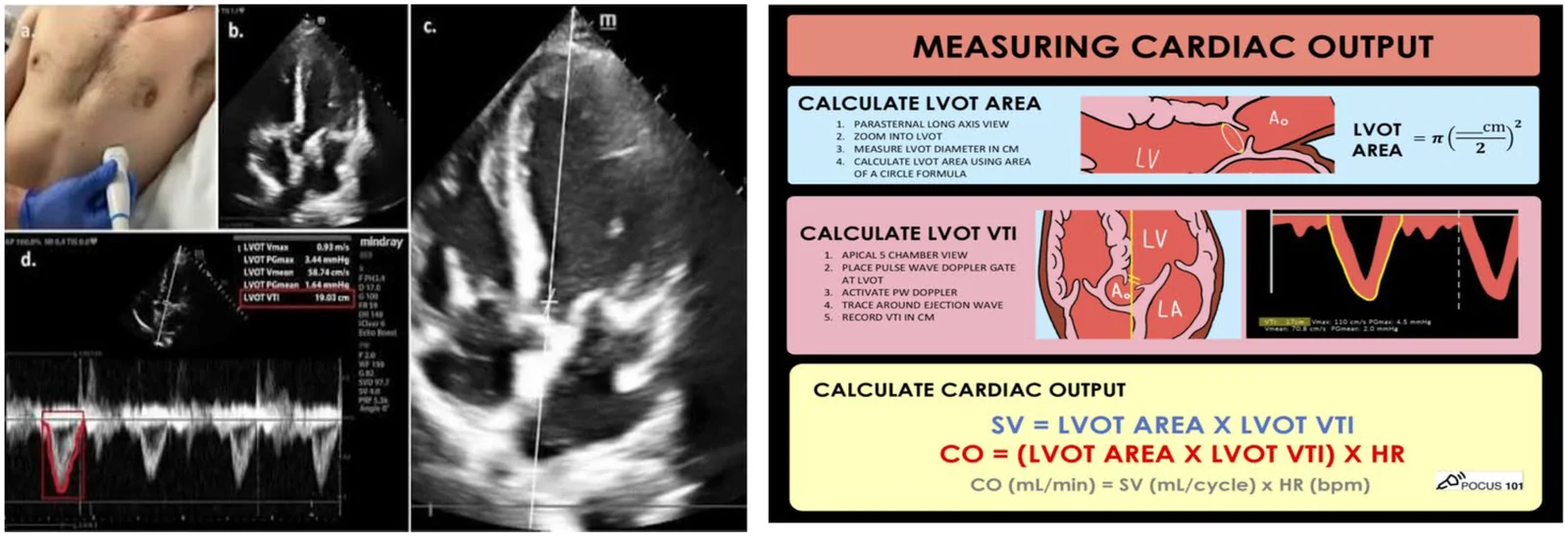
Calculating stroke volume by the velocity–time integral method. LVOT diameter is measured in the parasternal long-axis view and converted to area; LVOT VTI is obtained with pulsed-wave Doppler from the apical five-chamber view. Stroke volume is the product of the two, and cardiac output is stroke volume multiplied by heart rate.

Two practical points determine measurement quality. First, because LVOT area is derived from the square of the diameter, small errors in diameter measurement are amplified. For serial assessment within a single patient, this matters less than it might seem: LVOT area is fixed over the relevant timescale, so changes in VTI alone track changes in stroke volume. Tracking *Δ*VTI rather than recalculating absolute stroke volume at each time point removes the largest source of error (57).

Second, keep the Doppler angle of intonation as close to parallel with flow as possible, and place the sample volume consistently at the same location in the outflow tract on serial studies. Where image quality is inadequate, alternatives include right ventricular outflow tract VTI, mitral VTI, carotid or common femoral artery Doppler, and non-invasive bioreactance, which was the modality used in the FRESH trial (39). Transthoracic echocardiography has been validated against thermodilution with acceptable accuracy and precision in critically ill patients (58).

## 7 Stroke volume alone is insufficient: the dynamic challenge

A single stroke volume measurement, however accurate, does not answer the clinical question. A stroke volume of 55 mL may reflect a preload-dependent ventricle that will respond briskly to volume, or a failing ventricle already at the plateau of its Frank–Starling relationship. Distinguishing the two requires a preload perturbation and a repeat measurement (Figure 11).

**Figure 11:**
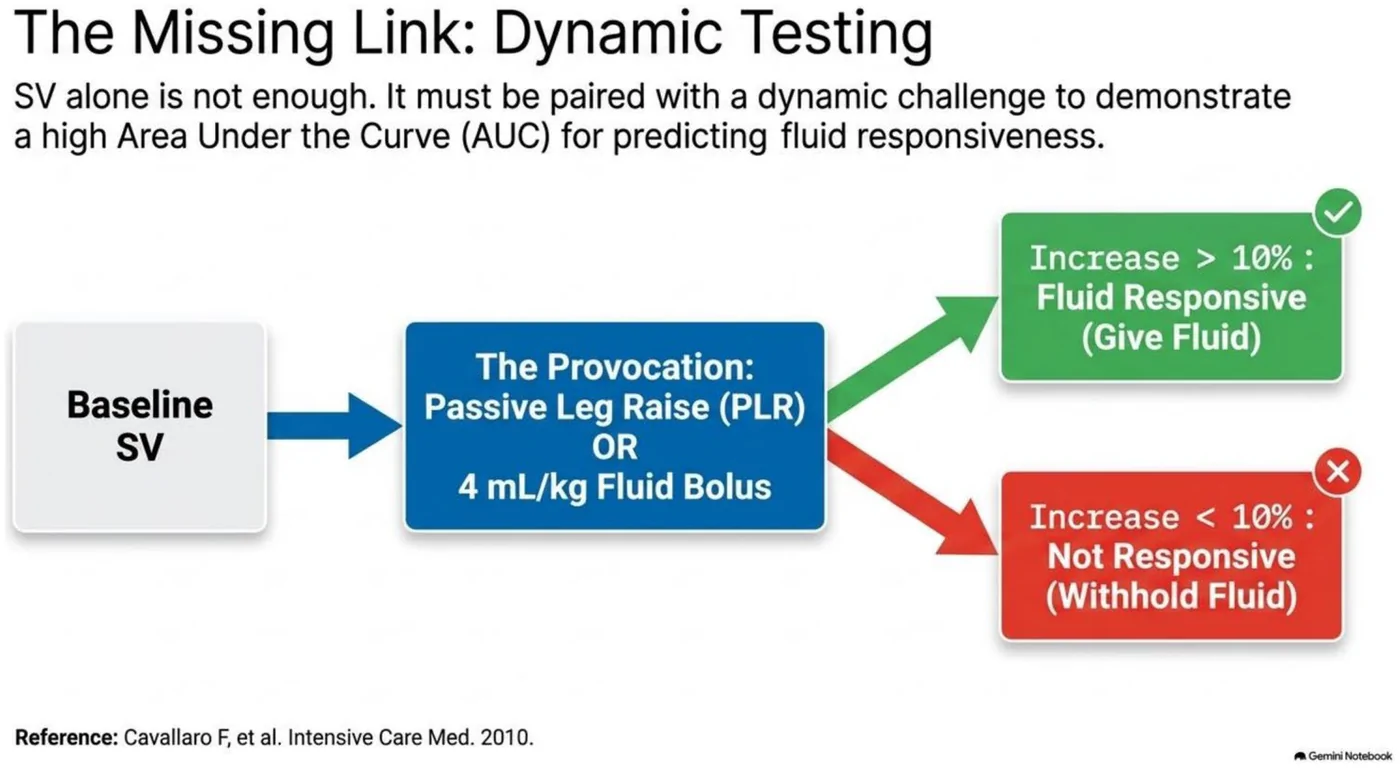
The dynamic testing algorithm. Baseline stroke volume is measured, a reversible preload challenge is applied, and stroke volume is remeasured. An increase above roughly 10% identifies a fluid responder; a smaller increase indicates that further volume will produce congestion without meaningful gain in cardiac output.

### 7.1 The passive leg raise

The passive leg raise (PLR) is the preferred challenge in septic shock because it is fully reversible. Transferring the patient from a semi-recumbent position to supine with the legs elevated to 45 degrees autotransfuses roughly 200–300 mL of venous blood from the splanchnic and lower limb reservoirs into the central circulation. If the patient does not respond, nothing has been given, and nothing must be removed.

Its diagnostic performance is among the best established in critical care. Cavallaro and colleagues pooled nine studies and reported a sensitivity of 89.4% (95% CI: 84.1–93.4) and a specificity of 91.4% (95% CI: 85.9–95.2) for PLR-induced changes in cardiac output, with a diagnostic odds ratio of 89.0 and an area under the ROC curve of 0.95. Critically for our index case, they concluded that this performance holds regardless of ventilation mode and cardiac rhythm (59). The larger meta-analysis by Monnet, Marik, and Teboul—21 studies and 991 patients—reproduced this, with a pooled positive likelihood ratio of approximately 11 and specificity above 90% when the effect was measured as a change in cardiac output. The same analysis found that assessing the PLR effect by change in pulse pressure alone preserves specificity but loses sensitivity and should be avoided when a stroke volume measurement is available (60).

The test is easy to perform. Monnet and Teboul's five rules remain the practical standard (61):

1. Start from a 45-degree semi-recumbent position, not supine, so that the splanchnic reservoir is recruited in addition to the legs.
2. Move the whole bed rather than lifting the legs manually, to avoid pain, awakening, and sympathetic stimulation that confound the result.
3. Measure a direct index of cardiac output or stroke volume, not blood pressure.
4. Measure within 30 to 90 s, because the effect peaks early and dissipates.
5. Confirm that stroke volume returns to baseline when the patient is repositioned, verifying that the perturbation, and not a coincident change, produced the effect.

PLR is unreliable or unsafe in a few settings: intra-abdominal hypertension above roughly 16 mmHg blunts the venous transfer, and elevated intracranial pressure is a contraindication (62). Given a BMI of 37, consider intra-abdominal pressure in our index case, and a persistently negative PLR in that context warrants confirmation with an alternative challenge.

### 7.2 Alternatives when PLR is unavailable

The mini-fluid challenge, 100 mL of crystalloid over one minute, with stroke volume measured immediately, is a reasonable substitute, though the smaller preload change requires a more precise measurement technique and a lower diagnostic threshold, typically around a 5%–6% rise in VTI (63, 64). The end-expiratory occlusion test, a 15 s interruption of ventilation that removes the cyclic impediment to venous return, performs well but requires a passively ventilated patient tolerant of the maneuver, which excludes our index case (64, 65). A 4 mL/kg bolus, as depicted in Figure 11, is also acceptable, but unlike the PLR it commits volume that cannot be retrieved.

### 7.3 Choosing a threshold

The conventional threshold is a 10% increase in stroke volume, with some protocols using 10%–15%. The precise cut-off matters less than recognizing that a grey zone exists around it: values between roughly 8% and 13% are indeterminate given the least significant change of most measurement techniques, and in that range the decision should be driven by fluid tolerance and by the overall trajectory rather than by the number alone (62, 66).

## 8 Assessing the other half: fluid tolerance

Establishing responsiveness answers only the cardiac question. A patient can be reliably responsive and still be the wrong candidate for volume. Tolerance assessment has matured considerably over the last five years, and two bedside tools now dominate.

### 8.1 Venous congestion and the VExUS approach

The venous excess ultrasound (VExUS) grading system integrates IVC diameter with pulsed-wave Doppler waveform morphology in the hepatic, portal, and intrarenal veins to grade systemic venous congestion (Figure 12). Progressive congestion produces a characteristic sequence: the hepatic vein waveform shifts from systolic-predominant to diastolic-predominant and ultimately to systolic inversion; portal vein flow becomes increasingly pulsatile, with a pulsatility index rising through 30% and beyond 50%; and intrarenal venous flow degrades from continuous to biphasic to monophasic diastolic-only (67).

**Figure 12:**
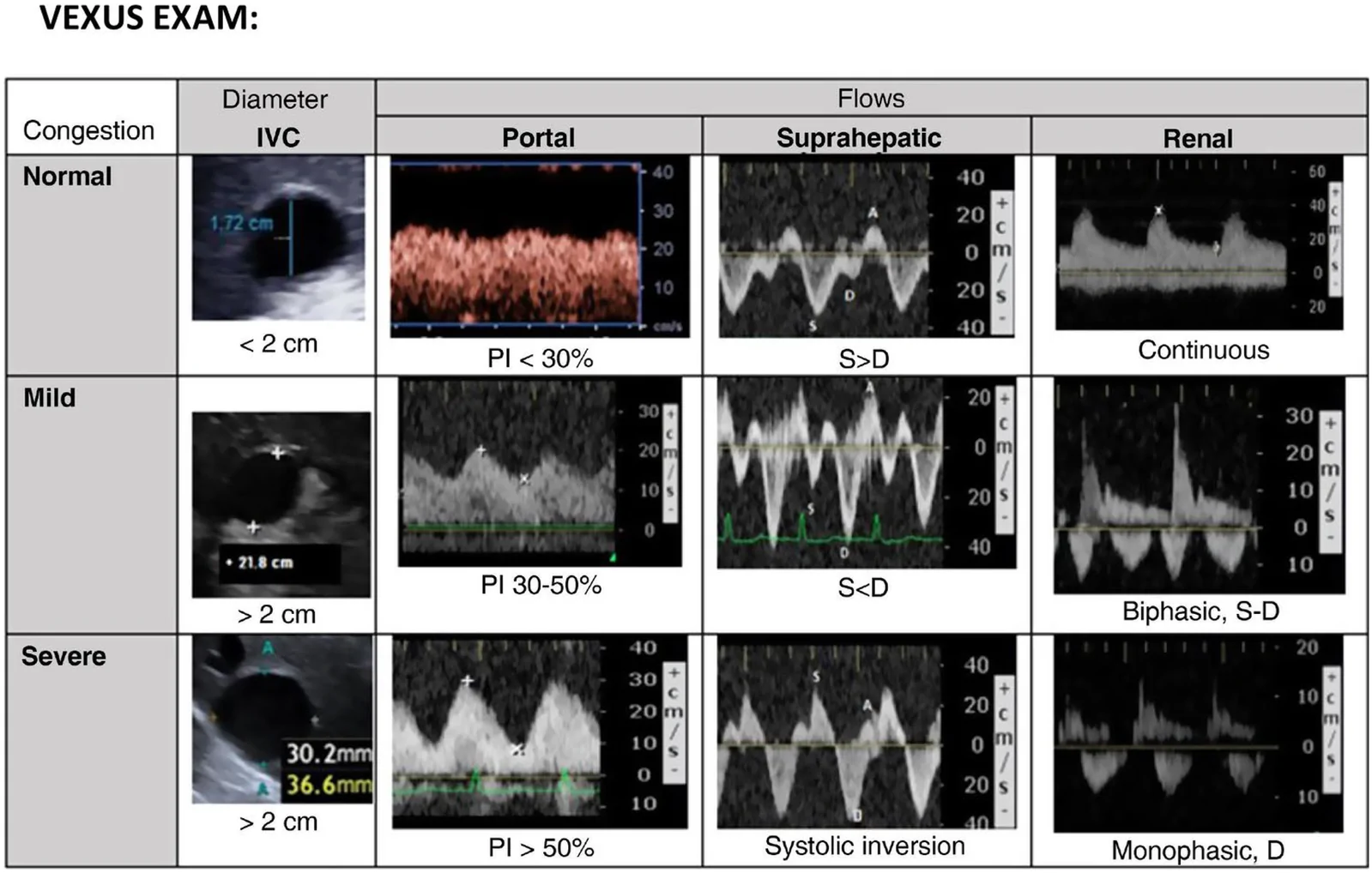
The VExUS examination. IVC diameter is combined with Doppler assessment of hepatic, portal, and intrarenal venous flow to grade congestion as normal, mild, or severe. Note that the IVC diameter component functions here as a marker of congestion rather than of fluid responsiveness—a different question with different validity conditions.

An important conceptual point is easy to miss. IVC diameter appears in both discussions but answers different questions. As an index of fluid responsiveness, it is unreliable in the conditions described in Section 5.1. As a component of a congestion assessment, a plethoric IVC combined with abnormal hepatic and portal Doppler carries meaningful information about downstream venous pressure, and this application does not depend on tidal volume or ventilatory mode. VExUS has been most extensively validated in cardiac surgical and cardiorenal populations, where higher grades predict acute kidney injury (67, 68); its performance in septic shock, where vasoplegia and altered venous compliance complicate interpretation, is less well established and should be regarded as an adjunct rather than a decision rule.

### 8.2 Pulmonary tolerance

Lung ultrasound provides complementary assessment. Progression from an A-line pattern to multiple bilateral B-lines indicates increasing extravascular lung water and is a practical marker of declining pulmonary tolerance (69). In a patient already requiring FiO_2_ 1.0 and PEEP 12 for bilateral infiltrates, this assessment is arguably redundant: the ventilator settings have already rendered the tolerance verdict. In less advanced disease, serial lung ultrasounds before and after a challenge can be informative.

Right ventricular function deserves specific attention. Sepsis-associated right ventricular dysfunction, compounded by high PEEP and hypoxic pulmonary vasoconstriction, converts volume loading into ventricular interdependence and reduced left ventricular filling. A dilated, hypokinetic right ventricle with septal flattening strongly argues against fluid, regardless of the PLR result.

## 9 Does precision fluid therapy change outcomes?

### 9.1 The FRESH trial

FRESH remains the most directly relevant randomized trial (Figure 13). One hundred and twenty-four patients presenting to emergency departments at 13 US and UK sites with sepsis-associated hypotension and anticipated ICU admission were randomized to PLR-guided stroke volume assessment using non-invasive bioreactance, performed before each fluid bolus or vasopressor escalation, vs. usual non-protocolized care. A stroke volume rise of at least 10%–15% prompted a fluid bolus; a non-response directed the clinician toward vasopressors instead (39).

**Figure 13:**
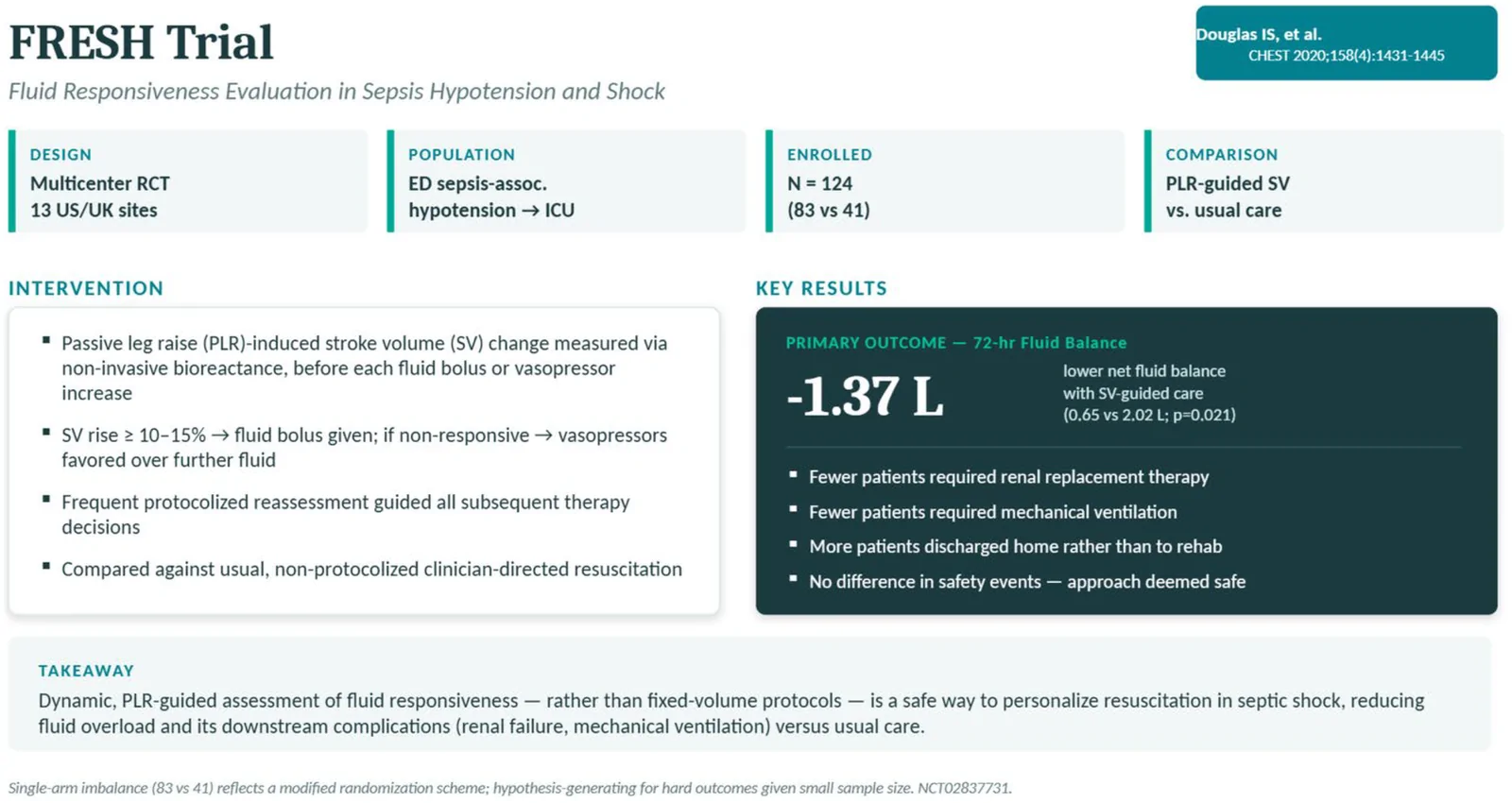
The FRESH trial. Dynamic, PLR-guided assessment of fluid responsiveness reduced 72 h net fluid balance by 1.37 L and was associated with less renal replacement therapy and less mechanical ventilation, with no excess of safety events. Douglas IS, et al. Chest. 2020;158(4):1,431–1,445 (39).

The intervention arm had a 1.37 L lower net fluid balance at 72 h (0.65 vs. 2.02 L; *P* = 0.021). Fewer patients required renal replacement therapy (5.1% vs. 17.5%) and fewer required mechanical ventilation (17.7% vs. 34.1%), both *P* = 0.04. More patients were discharged home rather than to a rehabilitation facility, and there was no excess of safety events (39).

These findings should be read with appropriate caution. FRESH enrolled 124 patients with an unequal allocation (83 vs. 41) reflecting a modified randomization scheme, and the organ support outcomes were secondary. The trial shows the approach is safe and meaningfully reduces fluid delivery; the hard outcome signals are hypothesis-generating.

### 9.2 Supporting cohort and meta-analytic evidence

Latham and colleagues reported a before-and-after cohort in which implementation of stroke-volume-guided resuscitation in severe sepsis and septic shock was associated with improved outcomes, providing implementation-level support for the approach (70).

Two meta-analyses address the question at a higher level. Bednarczyk and colleagues pooled 13 randomized trials enrolling 1,652 patients across mixed ICU populations and found that goal-directed therapy guided by dynamic assessment of fluid responsiveness was associated with a risk ratio for death of 0.59 (95% CI: 0.42–0.83; *I*^2^ = 0%), an absolute risk reduction of 2.9%, together with shorter ICU stay and shorter duration of mechanical ventilation (71). The principal limitation is that most included trials were perioperative rather than septic.

That gap has since been addressed. Wang and colleagues restricted their 2025 systematic review to randomized trials in sepsis and septic shock and found that dynamic measures of fluid responsiveness probably reduce 28-day mortality (RR: 0.61; 95% CI: 0.42–0.90; moderate certainty), may reduce acute kidney injury (RR: 0.66; 95% CI: 0.44–0.98; low certainty), and reduce day-3 cumulative fluid balance by a mean of 1.57 L (72). An earlier meta-analysis by Ehrman and colleagues reached a more skeptical conclusion, finding no mortality benefit, and the discrepancy reflects differing inclusion criteria and the accumulation of trials since 2019 (73).

### 9.3 What the guidelines say, and what they do not

The 2026 SSC guidelines suggest using dynamic measures to guide fluid resuscitation over physical examination or static measures alone, a conditional recommendation supported by low-certainty evidence. The accompanying remark defines dynamic measures as the response to a passive leg raise or a fluid bolus assessed by stroke volume, stroke volume variation, pulse pressure, or pulse pressure variation (1).

One further guideline statement deserves emphasis because it is frequently misread. The panel concluded that there is insufficient evidence to recommend for or against minimally invasive or non-invasive cardiac output monitoring in addition to usual care—but the accompanying remark specifies that this statement refers to devices requiring an arterial catheter or bioreactance-based systems, and that the use of critical care ultrasound was not evaluated (1). The absence of a recommendation for proprietary cardiac output monitors therefore does not argue against echocardiographic stroke volume assessment, which was outside the scope of the question posed.

## 10 Returning to the case

Applying this framework to the index case yields a clear answer and a clear rationale for rejecting the alternatives.

- Titration to IVC diameter is invalid. Spontaneous respiratory effort, a tidal volume of 6.35 mL/kg, and elevated intra-abdominal pressure from obesity each independently violate its prerequisites.
- Titration to PPV is invalid. Atrial fibrillation and a lung-protective tidal volume each disqualify it, and correcting either is not clinically appropriate here.
- Maintenance lactated Ringer's at 100 mL/h is unmonitored volume delivery amounting to over 7 L across 72 h in an anuric, hypoxemic patient. It is the least defensible of the four options.
- Titration to change in stroke volume, paired with a passive leg raise, remains valid across every one of this patient's physiological complications and is the appropriate choice.

A practical bedside sequence would be as follows. Obtain a baseline LVOT VTI averaged over at least ten consecutive cycles given the atrial fibrillation. Perform a PLR according to the five rules, moving the bed rather than the legs, and repeat the VTI within 60 to 90 s. If VTI rises by more than 10%, the patient is responsive—but the decision is not yet made. Assess tolerance: the FiO_2_ of 1.0 with PEEP 12 and bilateral infiltrates, together with a creatinine of 4.25 mg/dL, argues strongly against a large bolus even in a responder. A reasonable response would be a small, defined bolus of 250–500 mL with a repeat assessment, rather than an open-ended infusion, and a low threshold for escalating norepinephrine instead. If VTI rises by less than 10%, give no further fluid; prioritize vasopressor titration, inotropic support if echocardiography shows ventricular dysfunction, and reassessment of source control.

Two ancillary points are worth noting. First, this patient received 35 mL/kg in the emergency department. With a BMI of 37, the SSC 2026 remark on using adjusted or ideal body weight for the initial volume calculation means the effective dose relative to lean mass was higher still (1). Second, repeat the assessment. Fluid responsiveness is a state, not a trait; it changes over hours as capillary leak, vasopressor dose, and cardiac function evolve. A single PLR at ICU admission licenses one decision, not a management plan.

## 11 De-resuscitation

Once shock is resolved, the fluid question inverts. The 2026 SSC guidelines suggest active fluid removal in adults with septic shock after the acute resuscitation phase, using diuretics and, where diuretics are insufficient, ultrafiltration, with the decision informed by cardiorespiratory function, vasopressor dose, clinical trajectory, peripheral edema, weight, and fluid balance (1). Silversides and colleagues found in a meta-analysis that conservative or de-resuscitative fluid strategies after the resuscitation phase increased ventilator-free days without increasing adverse events (74).

The same measurement infrastructure serves both phases. A PLR that produces no rise in stroke volume in a patient with grade 2 or 3 venous congestion identifies someone in whom fluid removal is likely to be tolerated. Monnet and colleagues described this application, using PLR to guide fluid removal rather than fluid administration.

## 12 Limitations and open questions

Intellectual honesty requires acknowledging what remains unproven.

- The outcome evidence for dynamically guided resuscitation in sepsis rests on one modest, randomized trial, several before-and-after cohorts, and meta-analyses whose certainty is rated moderate at best. No large multicenter trial has been powered for mortality with a dynamic-guidance intervention in septic shock.
- Stroke volume measurement is operator-dependent. LVOT VTI requires training, adequate acoustic windows, and consistent technique; the method's smallest meaningful change sets a floor on the effect size that can be reliably detected.
- The 10% threshold is a convention derived from studies with heterogeneous reference standards, and a grey zone surrounds it.
- Fluid tolerance lacks a validated composite metric. VExUS is promising but has been studied mainly outside sepsis, and its grades have not been prospectively validated as decision thresholds in septic shock.
- Identifying a fluid responder does not establish that fluid is the right therapy. Many healthy volunteers are fluid responsive. Responsiveness is a necessary but not sufficient condition, which must be combined with evidence of hypoperfusion and evidence of tolerance.
- Bioreactance, pulse contour analysis, and echocardiography are not interchangeable, and trial results obtained with one modality may not transfer directly to another.

## 13 Conclusion

Over the last two decades, the trajectory has moved away from fixed fluid prescriptions and toward individualized titration. That shift is not primarily about giving less fluid, though less fluid is usually the result. It is about replacing assumptions with measurements.

For the practicing intensivist, three propositions follow. First, indiscriminate intravenous fluid is harmful in patients with ARDS and acute kidney injury, and the harm accumulates through maintenance infusions and drug diluents as surely as through boluses. Second, the physiology of the typical medical ICU patient- arrhythmia, lung-protective tidal volumes, spontaneous respiratory effort, obesity- invalidates pulse pressure variation and inferior vena cava assessment in the large majority of cases, and the prevalence of valid conditions for PPV is on the order of 2%. Third, stroke volume paired with a reversible dynamic challenge remains interpretable under all these conditions, and its use to guide resuscitation reduces fluid load, reduces the need for renal replacement therapy and mechanical ventilation, and probably reduces mortality.

The bedside discipline this implies is straightforward to state and demanding to sustain before every bolus: ask whether cardiac output will rise and whether the patient can tolerate the volume if it does. Measure, rather than guess, and measure again.
